# The Cellular and Molecular Interaction Between Erythrocytes and *Plasmodium falciparum* Merozoites

**DOI:** 10.3389/fcimb.2022.816574

**Published:** 2022-03-31

**Authors:** Jessica Molina-Franky, Manuel Elkin Patarroyo, Markus Kalkum, Manuel Alfonso Patarroyo

**Affiliations:** ^1^ Molecular Biology and Immunology Department, Fundación Instituto de Inmunología de Colombia (FIDIC), Bogotá, Colombia; ^2^ Department of Immunology and Theranostics, Arthur Riggs Diabetes and Metabolism Research Institute, Beckman Research Institute of the City of Hope, Duarte, CA, United States; ^3^ PhD Programme in Biotechnology, Universidad Nacional de Colombia, Bogotá, Colombia; ^4^ Health Sciences Division, Universidad Santo Tomás, Bogotá, Colombia; ^5^ Faculty of Medicine, Universidad Nacional de Colombia, Bogotá, Colombia

**Keywords:** malaria, host–parasite interaction, merozoite, invasion, pathogenesis, remodelling

## Abstract

*Plasmodium falciparum* is the most lethal human malaria parasite, partly due to its genetic variability and ability to use multiple invasion routes via its binding to host cell surface receptors. The parasite extensively modifies infected red blood cell architecture to promote its survival which leads to increased cell membrane rigidity, adhesiveness and permeability. Merozoites are initially released from infected hepatocytes and efficiently enter red blood cells in a well-orchestrated process that involves specific interactions between parasite ligands and erythrocyte receptors; symptoms of the disease occur during the life-cycle’s blood stage due to capillary blockage and massive erythrocyte lysis. Several studies have focused on elucidating molecular merozoite/erythrocyte interactions and host cell modifications; however, further in-depth analysis is required for understanding the parasite’s biology and thus provide the fundamental tools for developing prophylactic or therapeutic alternatives to mitigate or eliminate *Plasmodium falciparum*-related malaria. This review focuses on the cellular and molecular events during *Plasmodium falciparum* merozoite invasion of red blood cells and the alterations that occur in an erythrocyte once it has become infected.

## Introduction

Malaria caused by protozoa from the genus *Plasmodium* is the most serious and widespread parasitic disease of humans, involving 229 million cases and 409,000 deaths in 2019 (World Health Organization, 2021). At present, six members of *Plasmodium* species (*P. falciparum*, *P. vivax*, *P. malariae*, *P. knowlesi, P. ovale wallikeri and P. ovale curtisi*) have been shown to produce human malaria and all develop through the same general lifecycle which alternates between asexual stages in a human host and a sexual stage in an *Anopheles* mosquito (Moxon et al., 2020).

**Figure 1:**
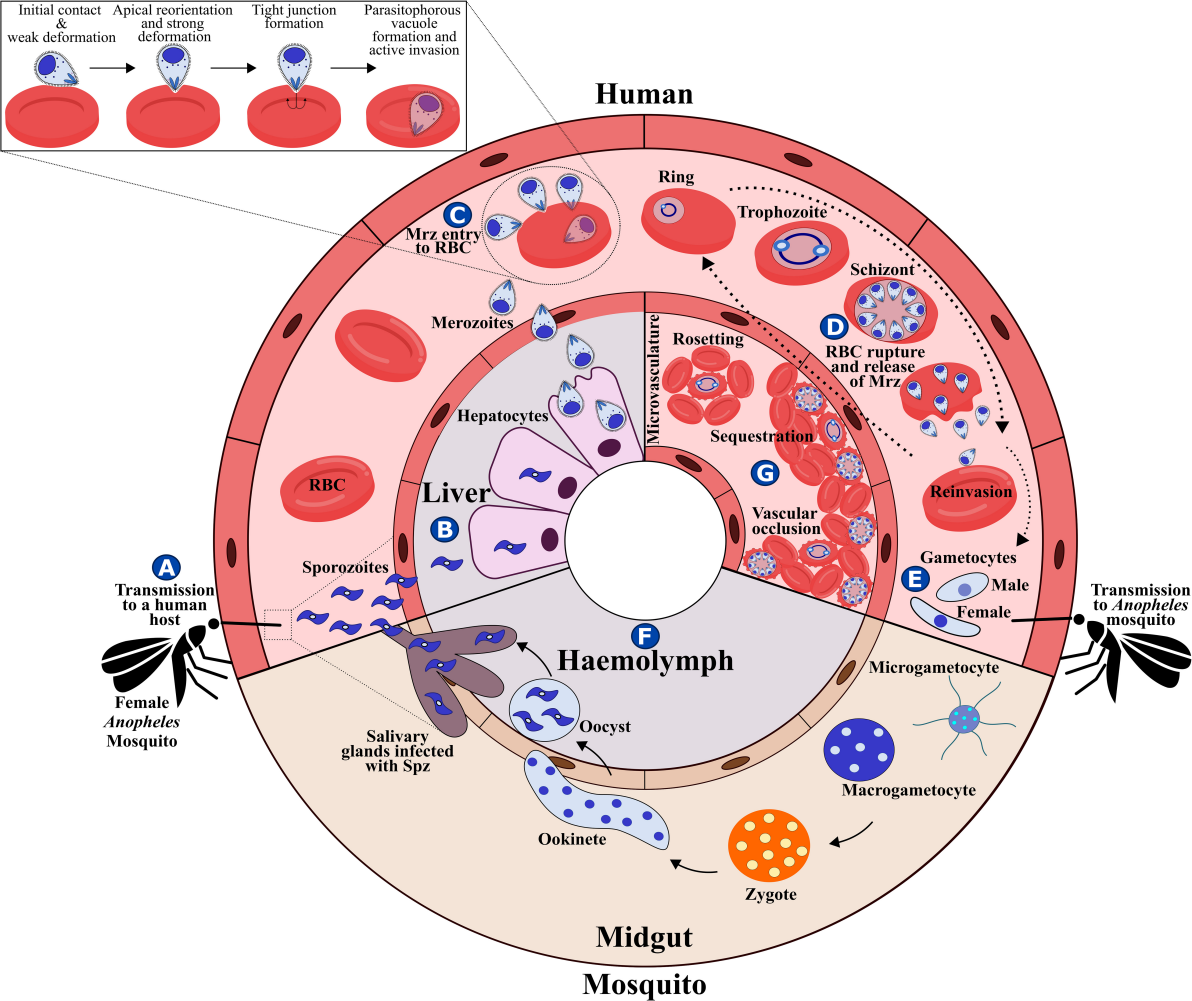
*P. falciparum* lifecycle. **(A)** *P. falciparum* Spz inoculation into a human host by a female *Anopheles* mosquito. **(B)** Spz entry to hepatocytes, Spz maturation into schizonts and Mrz release. **(C)** Mrz entry into RBCs. **(D)** Asexual multiplication within RBCs. **(E)** Some parasites differentiate to sexual stages; gametocytes are ingested by an *Anopheles* mosquito during a blood meal. **(F)** Sporogonic cycle. **(G)** Rosetting, sequestration and vascular occlusion.

**Figure 2:**
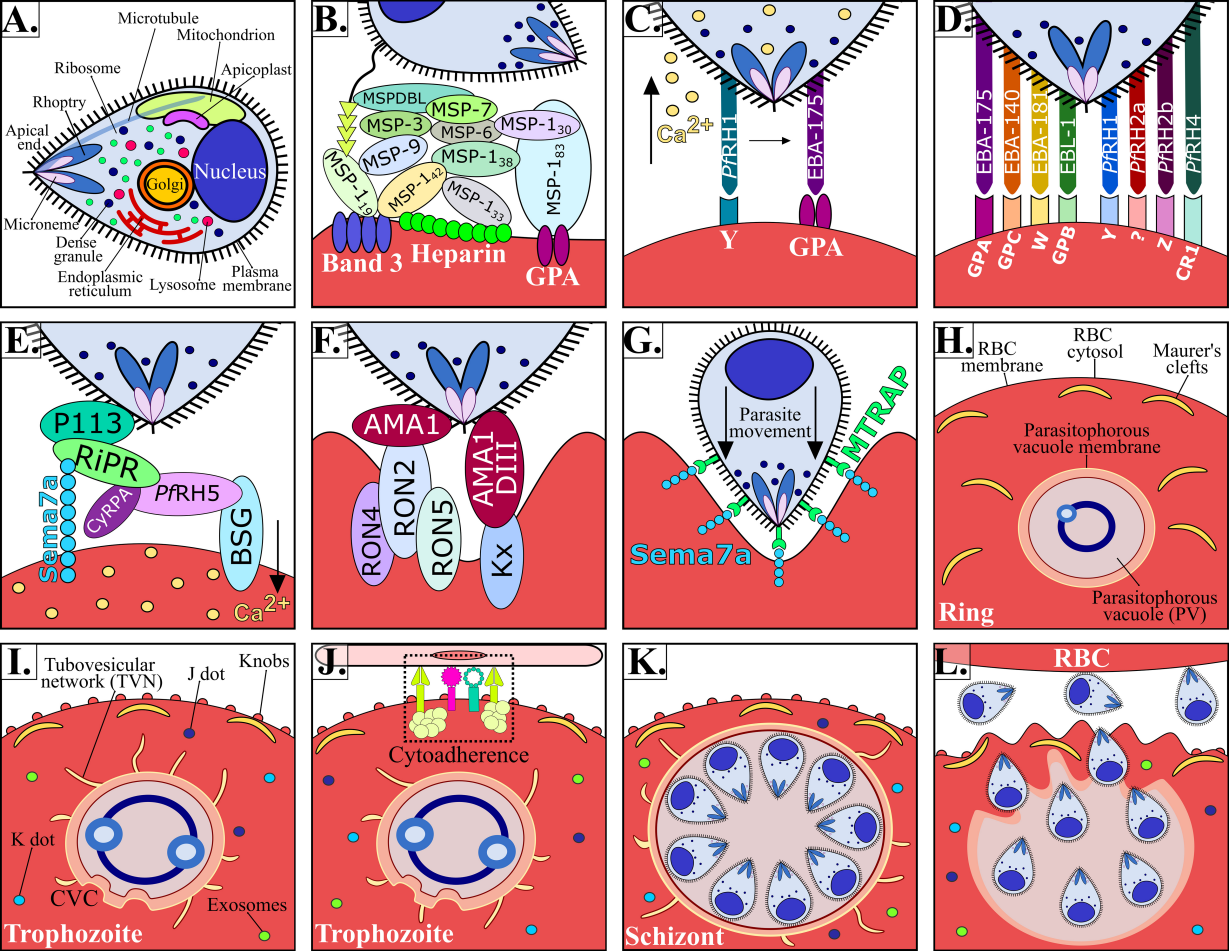
P*. falciparum* Mrz invasion and major changes induced in infected RBC. **(A)** *P. falciparum* Mrz **(B–G)** parasite ligands and RBC receptors involved in initial contact, apical reorientation and tight junction formation during Mrz invasion of RBC. **(H)** *P. falciparum* ring stage growing inside the PVM, representing the interface between parasite and host cytoplasm. Maurer’s clefts are flat and elongated membrane vesicles; they are mobile in iRBC cytoplasm during early parasite stages. **(I)** *P. falciparum* trophozoite stage. Maurer’s clefts are attached to membrane skeleton during mature parasite stages. Tubovesicular network (TVN) extending from the PVM into iRBC cytoplasm. Caveola vesicle complex (CVC) containing *P. falciparum* antigens which could be involved in the transport and release of specific parasite antigens from the iRBC and in plasma protein uptake. J dots, K dots and exosomes are membrane structures that traffic some parasite proteins (e.g. *Pf*EMP-1) through iRBC cytoplasm. Knobs covering the iRBC surface during late parasite stages. **(J)** *P. falciparum* cytoadherence mediated by the interaction between variant surface antigens and host receptors **(K)** *P. falciparum* schizont stage. **(K)** iRBC rupture and Mrz release. **(L)** Mrz release and RBC reinvasion.

The malaria parasite’s complex lifecycle starts when a female *Anopheles* mosquito bites and injects 10-100 sporozoites (Spz) into the human dermis (Hopp et al., 2019); Spz migrate into the bloodstream and invade hepatocytes in the liver. Each Spz develops into tens of thousands of merozoite (Mrz) forms within these cells (**Figures 1A, B**, **2A**) that are released into the bloodstream to invade red blood cells (RBCs) through specific interactions between *Plasmodium* parasite ligands and RBC receptors (**Figure 1C**, **2B–G** and **Table 1**) (Weiss et al., 2016). Once inside the RBC, the parasite self-encapsulates in the parasitophorous vacuole membrane (PVM) and immediately begins to remodel the host cell, causing internal and external modifications that enables it to survive and proliferate within RBCs. The parasite undergoes repetitive cycles of replication, exit and re-invasion every 48 hours, involving the parasite passing through ring, trophozoite and schizont forms (**Figure 1D**, **Figures 2H–L**). Symptoms associated with malaria coincide with massive RBC lysis during the lifecycle’s blood stage. A small number of developing Mrz differentiate into male and female gametocytes which are taken up by the mosquitoes during blood meals and mature into Spz in a mosquito’s midgut from where they migrate to its salivary glands to initiate another lifecycle (**Figures 1E, F**) (Moxon et al., 2020).

**Table 1:** *Plasmodium falciparum* receptor-ligand interactions.

Parasite ligand	RBC membrane receptors	Blood group system carried by the receptor	Kd	Receptor-ligand interface	Ref
MSP-1_19_	Band 3	Diego	ND	Band 3 720–761 aa bind to MSP1_19_ C-terminal	(Goel et al., 2003)
MSP-1_33_	GPA	MNS	ND	MSP-1 N-terminus	(Baldwin et al., 2015)
MSP-1_42_MSP-1_33_	Heparin	ND	ND	ND	(Boyle et al., 2010)
EBA-175	GPA	MNS	~0.26 μM	The dimer’s EBA-175 interface contains six GPA-binding glycans. K28, N417, R422, N429, K439 and D442 are the glycan 1 and 2 residues involved in binding, N33, N551, Y552, K553, M554 and N550 in glycans 3 and 4 and K28, N29, S32, R31, T340, K341, D342, V343, Y415, Y542 and Y546 in glycans 5 and 6.	(Duraisingh et al., 2003a; Tolia et al., 2005; Patarroyo et al., 2020)
EBA-140	GPC	Gerbich	ND	GPC residues 14-22 interact with EBA-140.	(Lobo et al., 2003)
EBA-181	“W” and Band 4.1	ND	ND	EBA-181 binds to the band 4.1 10 kDa fragment located between residues 404-471.W receptor binding is susceptible to NM and chymotrypsin treatment.	(Lanzillotti and Coetzer, 2006; Maier et al., 2009)
EBL-1	GPB	MNS	ND	The F2i peptide (residues ^601^C-V^669^) located in the DBL F2 sub-domain in EBL-1 contains the GPB binding site.	(Mayer et al., 2009; Li et al., 2012)
*Pf*Rh1	“Y”	ND	ND	Rh1 binds to a putative receptor called “Y” through a 333 aa fragment (500-833).	(Triglia et al., 2009)
*Pf*Rh2b	“Z”	ND	ND	Rh2b binding is trypsin and NM resistant and sensitive to chymotrypsin.	(Duraisingh et al., 2003b)
*Pf*Rh4	CR1	Knops	2.9 ± 0.2 μM	D^18^ and F^20^ located in CR1 LHR-A binds to *Pf*Rh4 N^328^-D^588^ The *Pf*RH4 30 kDa fragment (328N–D588) binds strongly to neuraminidase-treated erythrocytes.	(Gaur et al., 2007; Tham et al., 2010; Park et al., 2014)
*Pf*Rh5	BSG	Diego	1.12 ± 0.09 μM	*Pf*Rh5 F^350^, R^448^, T^449^, W^447^, N^354^, R^357^, S^197^, D^207^, Y^200^ and E^362^ bind to BSG T^25^, V^26^, T^28^, N^98^, E^84^, Q^100^, H^102^, K^191^, V^131^, S^190^, P^133^ and Q^164^.	(Chen et al., 2014; Galaway et al., 2017; Wong et al., 2019; Patarroyo et al., 2020)
*Pf*Ripr	Sema-7A	John-Milton-Hargen	9.4 x 10^-10^ M	*Pf*Ripr_5 ^720^C-D^934^ binds to SEMA-7a	(Nagaoka et al., 2020)
MTRAP	Sema-7A	John-Milton-Hargen	1.18 ± 0.40 μM	Two MTRAP monomers interact with the entire Sema-7A e ctodomain *via* their tandem thrombospondin type-I repeat (TSR) domains.	(Bartholdson et al., 2012)
AMA-1	Kx	Kx	ND	AMA-1 domain III binds to erythrocyte membrane transport protein Kx.	(Kato et al., 2005)

The *P. falciparum* parasite has different organelles, including a nucleus, a single mitochondrion, an endoplasmic reticulum and a rudimentary Golgi apparatus. It has novel organelles, such as apicoplast, a modified lysosome (digestive vacuole), and apical organelles (rhoptries, micronemes, dense granules and exoneme) involved in the entry to RBC (**Figure 2A**); however, an infected RBC is devoid of organelles and cellular machinery (Hanssen et al., 2010). Successful *P. falciparum* growth and division inside RBC therefore requires structural, biochemical and functional modifications to facilitate communication with the extracellular environment (Jonsdottir et al., 2021).

*P. falciparum* is considered the most lethal human malaria species owing to avoidance of splenic clearance of infected RBCs (iRBC) and by iRBC sequestration in the microvasculature of the brain and other vital organs (**Figure 1G**); such a process is not common for RBCs infected by other human malaria species. *P. falciparum* has an ability for antigenic variation and polymorphism to evade the human immune response and uses many invasion routes involving multiple redundant ligands interacting with host-specific receptors to invade RBCs (**Figures 2B–G** and **Table 1**) (Gilson et al., 2017).

This review focuses on describing the cellular and molecular events during *P. falciparum* Mrz invasion of RBCs and the alterations that occur in erythrocytes once they have become infected, contributing to a better understanding of the parasite’s biology and pathogenesis, highlighting possibilities for developing drugs and vaccines against this parasitosis.

## 2 The Uninfected Red Blood Cell Membrane

Uninfected RBC (uRBC) properties should be discussed for better understanding modifications made to RBC membrane as a consequence of the interaction with the parasite. Erythrocytes are mature RBCs; during erythropoiesis they lose their nucleus and the ability to synthesise new proteins (Mei et al., 2021). *P. falciparum* therefore infects host cells that mainly lack biosynthetic pathways and are bare of intracellular compartments, metabolically slow, and nutritional deprived.

RBC are a “sack” of haemoglobin contained by a phospholipid bilayer membrane having specialised tasks regarding oxygen and carbon dioxide transport between the lungs and the tissues through the circulatory system (Daniels, 2007). RBC can change their shape to fit through the microvasculature’s narrow capillaries to maintain structural integrity. This mechanical resilience is due to their membrane-associated protein skeleton which provides RBC with elasticity, flexibility and cytoplasmic viscosity that depends on intracellular ion, their haemoglobin concentration (~15g/dL) and surface to volume ratio (Depond et al., 2020). The balance between these parameters becomes disrupted during malarial infection.

**Figure 3:**
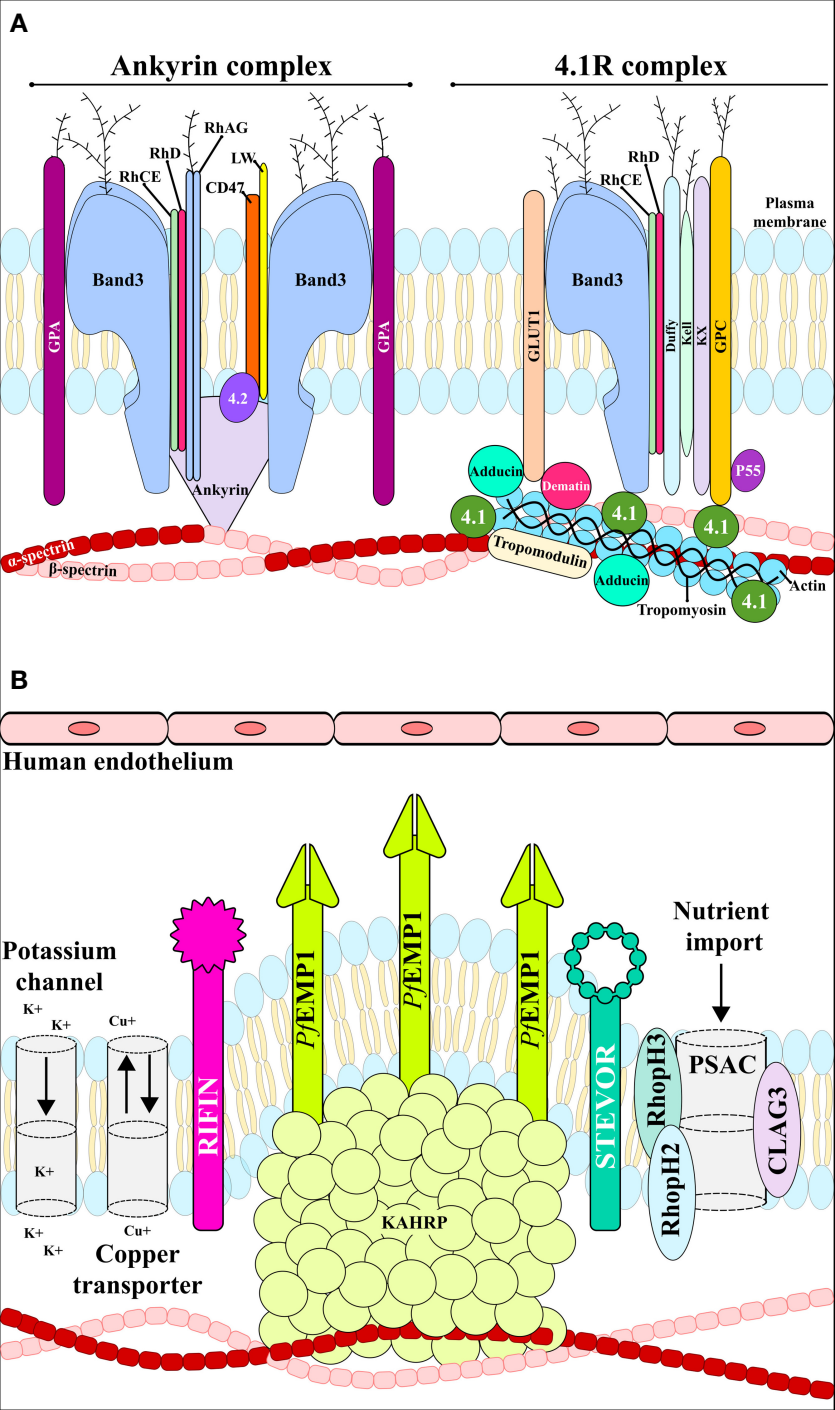
RBC membrane. **(A)** uninfected RBC membrane with the ankyrin and 4.1R complexes linking the bilayer with the membrane skeleton. **(B)** Infected RBC membrane with anion channels, the exported parasite proteins *Pf*EMP1, STEVOR and RIFIN, and the expressed adhesin KAHRP. These proteins interact with host receptors mediating cytoadherence to the human endothelial cells.

The plasma membrane is composed of a lipid bilayer with ~20 major and ~850 minor embedded proteins. These are mainly integral membrane, membrane-associated, GPI-anchored, extracellular and cytoskeletal proteins which are involved in protein binding, transport signal transduction, glycolysis, catalytic, structural and antioxidant functions. Structural integrity is provided by the bilayer linking to the membrane skeleton *via* a series of vertical interactions with different membrane proteins, forming the ankyrin and the 4.1R macro protein complexes (**Figure 3A**) (Mankelow et al., 2012).

The RBC cytoskeleton consists of a matrix mainly consisting of spectrin, actin, protein 4.1, ankyrin and actin-associated proteins (Lux, 2016). Ankyrin is a large protein (210kDa) featuring a central spectrin-binding domain, a carboxy-terminal regulatory domain and an amino-terminal domain that interacts with the β-spectrin head region and the band 3 residues 175−185 and 63−73 (Chang and Low, 2003; Grey et al., 2012). This interaction promotes ankyrin tetramerisation, thereby decreasing its diffusional mobility. Interaction with protein 4.2 and Rhesus proteins helps to stabilise the vertical interaction (**Figure 3A**). The 4.1R protein forms a complex with actin and spectrin which defines the membrane-skeletal network’s nodal junctions and interacts with glycophorin C (GPC), band 3, Kx, Rh, Duffy, Kell and p55. The RBC glucose transporter (GLUT1) interacts with adducin and dematin forming a bridge between the protein network and the membrane bilayer, providing a second point of attachment to the plasma membrane (**Figure 3A**) (Salomao et al., 2008). Most of these membrane proteins are of critical importance to the *P. falciparum* intra-erythrocyte phase by interacting with the ligands and providing a gateway to host cells during invasion (**Figures 2B–G** and **Table 1**).

## 3 Plasmodium Falciparum RBC Invasion

*P. falciparum* Mrz invasion of RBCs is a well-orchestrated process, which is mediated by Mrz ligand interaction with RBC proteins through several distinct steps including initial contact, apical reorientation, tight junction (TJ) formation and active invasion (**Figures 1C**, **2B–G**) (Weiss et al., 2016).

### 3.1 Initial Contact

Initial interaction is a crucial step involving Mrz passing over an RBC membrane through non-specific, reversible, low affinity interactions leading to slight deformation of RBC surface by increasing the area of ​​contact with the Mrz (**Figure 2B**) (Weiss et al., 2016). Initial contact is mediated by Mrz surface proteins 1 to 10 (MSP-1 to MSP-10), most of these (due to their location) are exposed to the host’s immune system, therefore having highly variable sequences; their conserved regions are crucial for establishing initial interactions with host cells, representing a highly promising target for antimalarial vaccine development (Beeson et al., 2016). MSP-1, MSP-2, MSP-4, MSP-8 and MSP-10 are bound to the Mrz membrane through glycosylphosphatidylinositol (GPI) anchors (Sanders et al., 2006). MSP-3, MSP-6, MSP-7 and MSP-9 are soluble and weakly bound to the Mrz surface or in association with other membrane proteins, forming high weight molecular complexes with a complex degree of interaction between them (**Figure 2B**) (Cowman et al., 2012; Beeson et al., 2016). These proteins are processed during host cell invasion; the GPI fragments anchored to the Mrz membrane are the only ones that penetrate the RBC, i.e. making them a target for vaccine development.

#### 3.1.1 MSP-1 - Glycophorin A – Band 3 - Heparin-like molecules

MSP-1 is the most abundant Mrz surface protein; it consists of 1,521 amino acids (aa) and has a ~190 kDa molecular weight. This protein is the most important ligand involved in the initial host cell contact. It undergoes proteolytic processing when 8-24 Mrz are released during schizont rupture, creating four fragments (83, 30, 38 and 42 kDa) which remain as a multi-subunit complex on the Mrz surface. The MSP-1_42_ fragment undergoes a second Ca^2+^-dependent cleavage resulting in the formation of 33 and 19 kDa fragments. Most of these fragments are shed from the Mrz surface during invasion; however, MSP-1_19_ remains attached (**Figure 2B**) (McBride and Heidrich, 1987).

MSP-142 and MSP-1_33_ fragments binds to heparin-like molecules (**Figure 2B** and **Table 1**), located on the surface of the RBCs. Heparin-like molecules are involved in different biological process such as cell proliferation, angiogenesis, immune system modulation, cell migration and cellular invasion (Boyle et al., 2010). It has been suggested that heparin can inhibit the schizont rupture and/or Mrz invasion **(** Butcher et al., 1988 **;** Kisilevsky et al., 2002 **).** In presence of 100 μg/mL of heparin, the Mrz invasion of *P. falciparum* W2mef (sialic acid–dependent invasion phenotype) and 3D7 (sialic acid–independent invasion phenotype) was inhibited by ~80% (Boyle et al., 2010).

It has also been found that MSP-1 binds to RBC membrane through MSP-1_83_ N-terminal interactions in which glycophorin A (GPA) extracellular domain and MSP-1_42_ interact with two band 3 non-glycosylated extracellular regions (aa 720-761 and 807-826) (**Figure 2B** and **Table 1**) (Goel et al., 2003; Baldwin et al., 2015).

GPA is a transmembrane protein and is one the most abundant proteins on the RBC membrane (1x10^6^ copies per cell). It consists of 150 aa (72 in the extracellular domain, followed by 23 in the transmembrane domain, and 36 in the intracellular domain) and N-glycosidic side chains on Asn^26^. GPA has been described amongst the main antigenic determinants for MN and Ss blood groups; it is involved in RBC interaction with the vascular endothelium (Tomita and Marchesi, 1975).

Band 3 is a 911 aa glycoprotein having 110-kDa molecular weight. It is the most abundant protein (25%) on the RBC membrane and is an essential RBC receptor in *P. falciparum* sialic acid-independent invasion pathways (Goel et al., 2003). Band 3 has two domains: a cytosolic N-terminal domain (aa 1-360) and an integral membrane domain containing 8 transmembrane helices (aa 361 to 911). The C-terminal domain catalyses anion exchange (Cl^-^ and HCO^-^) through the RBC membrane, thereby increasing its CO_2_ transport capacity. This domain is associated with carbon anhydrase II which plays a role in CO_2_ transport from the tissues to pulmonary circulation. The cytoplasmic N-terminal domain is an important anchoring site for other membrane-associated proteins, such as ankyrin, deoxyhaemoglobin, protein 4.1, protein 4.2, glyceraldehyde-3-phosphate dehydrogenase, aldolase, protein tyrosine kinase and phosphofructokinase (**Figure 3A**) (Arakawa et al., 2015, 3).

Band 3 is associated with GPA through its eight transmembrane helix, forming a dual protein, thus producing the so-called Wrb blood group antigen (Baldwin et al., 2015). This association can increase the probability of adhesion of the parasite ligands involved in contact with host cells. All the above indicates an important role for the MSP-1-band 3/GPA complex formation during the initial adhesion phase of RBC invasion by *P. falciparum*.

Fine mapping of MSP-1 RBC interaction points has led to the determination of 20 aa-long peptides (called high activity binding peptides - HABP). MSP-1 HABPs identified to date include 1513 (^42^G-A^62^) and 1522 (^202^Q-V^221^) located in the 83 kDa fragment, 1577 (^1142^F-K^1161^) and 1582 (^1242^A-I^1261^) situated in the 38 kDa fragment, 1585 (^1302^E-E^1321^), 1589 (^1382^L-D^1401^), 1590 (^1402^V-I^1421^) and 1591 (^1422^K-I^1441^) located in the 33 kDa domain and 5501 (^1542^Q-S^1561^) in the 19-kDa fragment (Urquiza et al., 1996). A portion of the HABP 1513 sequence was included in the only chemically-synthesised vaccine produced so far against malaria (S*Pf*66); it induced 30%-50% protective efficacy in several clinical trials (Patarroyo et al., 1987).

Anti-*Pf*MSP-1_19_ monoclonal antibody (mAb) 12.10 inhibits *P. falciparum* Mrz invasion of RBCs by 72% (T9-94 strain) and 50% (T9-96 strain), respectively (Blackman et al., 1990). Mice immunised with three 10 μg doses of the vaccine *Pf*MSP 1/8 induced high anti-*Pf*MSP1_19_-specific IgG titres, inhibiting the *in vitro* growth of *P. falciparum* FVO and 3D7 strains by 80% (Alaro et al., 2013).

MSPs are promising anti-malarial vaccine candidates due to their location on the parasite and their exposure to the host**’**s immune system. A recent single-centre, randomised, double-blind, phase 1a clinical study using the full-length *P. falciparum* MSP-1 in combination with GLA-SE adjuvant was carried out to evaluate its safety and immunogenicity in 32 volunteers vaccinated at least three times. The vaccine was well-tolerated and immunogenic; MSP-1-specific IgM and IgG titres persisted above levels found in malaria semi-immune humans for at least 6 months after the last dose. The antibodies (Abs) were variant and strain transcending and stimulated respiratory activity in granulocytes. The MSP-1 vaccine induced memory T-cells (Blank et al., 2020).

However, difficulties in obtaining protective immunity with MSP-1 fragments have been described due to this protein**’**s large amount of dimorphic and/or polymorphic sites. Such diversity seems to be associated with the parasite**’**s immune system evasion mechanisms (Rodriguez et al., 2008).

### 3.2 Apical Reorientation

The Mrz reorient themselves, bringing their apical poles into contact with erythrocyte membrane, establishing specific and high binding affinity interactions mediated by ligands from the erythrocyte-binding like (EBL) or the reticulocyte-binding homologous protein (*Pf*Rh) families, creating a strong deformation of the RBC membrane (**Figures 2C–G**) (Weiss et al., 2016).

Members of the EBL family are transmembrane type 1 proteins located in Mrz apical pole micronemes; they include erythrocyte binding antigens 175, 140 and 181 (EBA-175, EBA-140/BAEBL, and EBA-181/JESEBL), erythrocyte binding ligand 1 (EBL-1) and Mrz adhesive erythrocytic binding protein (MAEBL) (Tham et al., 2012). Their amino acid sequences consist of signal sequences, followed by six regions (RI-RVI) containing cysteine-rich (Cys-rich) fragments and highly conserved aromatic residues between orthologues**’** RII and RVI where RII consists of one or two Duffy binding-like (DBL) domains (including the receptor binding domain), splitting into regions F1 and F2. Regions IV-VI are followed by a transmembrane region and a small cytoplasmatic tail anchoring EBLs to the Mrz membrane (Tham et al., 2012).

EBL proteins are important for RBC invasion and bind to RBC in a sialic acid-dependent manner (Cowman et al., 2017); it has been shown that neuraminidase (NM) treatment removes sialic acid from RBC glycoproteins and reduces Mrz invasion nine-fold (Weiss et al., 2015).

The RH family comprises *Pf*RH1, *Pf*RH2a, *Pf*RH2b, *Pf*RH3, *Pf*RH4 and *Pf*RH5, however, *Pf*RH3 has missense mutations and is a pseudogene which is transcribed but not translated during any of the parasite**’**s life-cycle stages (Ord et al., 2015). These proteins have high molecular weight and are homologues of the *P. vivax* reticulocyte binding protein (*Pv*RBP-1 and -2) and *P. yoelii Py*235 (Taylor et al., 2001). Protein structures are similar for *Pf*RH1, *Pf*RH2a, *Pf*RH2b and *Pf*RH4, containing a signal peptide, a homologous region in RH and a transmembrane domain (except for *Pf*RH5 which lacks this domain) (Wright et al., 2014).

Ca^2+^ levels in the Mrz may combine different signals from the environment during invasion, mimicking extracellular conditions. Low K^+^ concentration leads to increased Mrz intracellular Ca^2+^ and EBA-175 release, translocating it from the Mrz membrane (Singh et al., 2010). However, EBA-175 release requires *Pf*Rh1 interaction with its RBC receptor in a K^+^-independent manner (Gao et al., 2013), followed by interaction with any EBL family or *Pf*Rh family redundant proteins, thereby enabling *Pf*Rh5 binding to basigin (BSG), triggering a Ca^2+^ signal in the RBC causing changes in RBC cytoskeleton architecture and protein phosphorylation (**Figures 2C–E**). EBA-175, EBA-140, EBA-181, EBL-1, *Pf*RH2a, *Pf*RH2b and *Pf*RH4 are known to be alternative pathway ligands since they appear to be functionally redundant, their expression depending on receptor characteristics and Ab preformed from previous infections. However, it has been suggested that all of these proteins work together when their functions are combined (Weiss et al., 2015).

#### 3.2.1 EBA-175 – Glycophorin A

EBA-175 consists of 1,470 aa, has a 175 kDa molecular weight and binds GPA (the major RBC sialoglycoprotein); such binding is facilitated by highly conserved RII formed by Cys-rich regions F1 (residues 8-282) and F2 (residues 297-603). These are *P. vivax* Duffy binding protein homologues and have thus been referred to as Duffy binding-like (DBL) domains (Adams et al., 1992). GPA was the first receptor on RBCs identified as a *P. falciparum* invasion ligand and its binding to EBA-175 restores Mrz cytoplasmic Ca^2+^ base levels and triggers rhoptry protein release (**Figures 2C, D**) **(** Singh et al., 2010 **).** This interaction was characterised by being resistant to chymotrypsin**’**s enzymatic action and is susceptible to NM, which cleaves the sialic acids attached to glycophorin**’**s oligosaccharide α side-chains (Duraisingh et al., 2003a).

Tolia**’**s X-ray crystallography studies (the first to involve *P. falciparum* proteins) of the EBA-175 RII recombinant fragment (analysed at 2.3 Å resolution) have shown it to be a homodimer in which two RII molecules are arranged in an anti-parallel fashion, interacting in a “handshake-like” manner. The centre of the homodimer has two channels. Most of the residues forming the surface of both channels belong to two of the dimer**’**s F2 domains. The dimer**’**s interface has six glycan binding sites (four located within the channel and two exposed in a cavity on the external surface). The six glycans are in contact with residues from both RII monomers, indicating that EBA-175 dimerisation is functionally important for receptor-ligand interaction and Mrz invasion. RII was co-crystallised with α-2,3-sialyllactose glycan for further characterisation of the EBA-175 RII receptor-binding interaction with GPA. The Neu5Ac(α2,3)-Ga sialic acid component within the glycan was found to be required for this interaction (Tolia et al., 2005).

Nevertheless, other studies using flow cytometry and ELISA have provided evidence that EBA-175 may also bind to desialylated GPA by engaging a 21 aa fragment (aa 1076–1096) lying between RIII and IV (Jakobsen et al., 1998; Healer et al., 2013).

HABPs 1758 (^80^K-N^99^) located in RI, 1779 (^500^N-I^519^) and 1783 (^580^H-K^599^) located in RII, 1815 (^1220^Y-H^1239^) and 1818 (^1280^N-L^1299^) located in RIV have been identified for EBA-175 (aa positions based on the *P. falciparum* Camp/Malaysia strain). HABPs 1779 and 1783 were located in EBA-175 RII-GPA binding region positions (^356^N-I^375^) and (^436^H-K^455^) after aligning EBA-175 sequences from the Camp/Malaysia strain and 3D7 used by Tolia (Rodriguez et al., 2000; Tolia et al., 2005). HABP 1758 inhibited parasite invasion by 85%, 1779 by 24% and 1783 by 34% (Rodriguez et al., 2000).

Some mAbs (i.e. R215, R217 and R256) are specific and interact with F2**’**s RII domain, and potently block EBA-175 binding to RBC, preventing Mrz invasion; mAb R216 binds F2 to a lesser extent but cannot effectively block the protein**’**s binding to RBCs. Also, mAb R218 binds to the F1 domain, and manages to inhibit parasite growth with less efficacy than the F2-specific Abs (Sim et al., 2011). Interestingly, anti-RII Abs can block sialic acid-dependent invasion pathways and inhibit alternative sialic acid-independent ones (Narum et al., 2000). A study using naturally-acquired human Abs against EBA-175 RII from Senegalese individuals reported 20%-50% inhibition of parasite invasion which was not sialic acid-dependent (Badiane et al., 2013). The results suggested that the parasite used multiple ligand-receptor interaction pathways and it was found that *P. falciparum* Mrz invasion became inhibited by around 60%-80% using anti-conserved EBA-175 RIII-V Abs. EBA-175 RII-V should thus be considered a multi-component malaria vaccine candidate (Healer et al., 2013).

#### 3.2.2 EBA-140 – Glycophorin C

EBA-140 (also known as BAEBL or *Pf*EBP-2) is an EBA-175 paralogue, sharing structural homology regarding DBL domains and including RII within its F1 and F2 homologous domains (Adams et al., 1992).

EBA-140 binds to GPC (**Figure 2D** and **Table 1**), a protein expressed in 135–143 x10^3^ copies on the RBC membrane (Lobo et al., 2003, 2; Satchwell, 2016); it has an extracellular transmembrane and cytoplasmatic domains distributed in 128 residues, having 12 glycans bound to O and a single glycan ligated to N. This glycophorin performs an important function in maintaining RBC shape and regulating cell membrane components through interaction with protein 4.1 (**Figure 3A**) (Satchwell, 2016).

A region in GPC from aa 11 to aa 24 interacts with EBA-140 (Lobo et al., 2003, 2; Maier et al., 2003). X-ray crystallography of RII (^148^R-E^740^) determined EBA-140 as a monomer; however, it is not clear whether interaction with the receptor produces a multimer assembly, similar to what occurs when EBA-175 is bound to GPA (Tolia et al., 2005; Lin et al., 2012). Functional and structural studies have shown that the F1 and F2 domains have highly conserved sialic acid binding sites; furthermore, receptor binding was determined by sialic acid binding to F1 (Malpede et al., 2013).

The fine mapping of the interaction between EBA-140 and RBC identified HABPs 26139 (^301^D-S^320^), 26144 (^401^D-M^420^), 26147 (^461^L-L^480^), 26160 (^861^G-Y^880^) and 26170 (^1061^C-P^1080^) (Rodriguez et al., 2003). 26139, 26144 and 26147 lie in RII, directly interacting with GPC (Rodriguez et al., 2003). Studies have shown that ^416^Y (included in HABP 26144) established H-bonds with the GPC glycan 2 sialic acid acetamide group in which this residue**’**s mutations reduced RBC binding capacity by 35.2% (Kobayashi et al., 2010; Malpede et al., 2013).

It is known that some EBA-140 variants can bind NM-treated RBCs, thereby suggesting that GPC is not the receptor for them (Maier et al., 2009). Altogether, the data suggests that *P. falciparum* invasion proteins have redundant functions since EBA-140 is not essential for Mrz invasion of RBC (as long as sufficient similar invasion ligands interact with host cell receptors); moreover, EBA-140 is not expressed in all *P. falciparum* strains (Lobo et al., 2003, 2).

#### 3.2.3 EBA-181 – W

EBA-181 is encoded on *P. falciparum* chromosome 1. It is 1,567 aa-long and has a 181 kDa molecular weight; it is located in the micronemes and is co-expressed with EBA-175 (Gilberger et al., 2003). Its primary structure consists of a hydrophobic N-terminal region with two Cys-rich regions (F1–F2) in the extracellular domain and a Cys-rich domain in the C-terminal region, a short transmembrane domain and cytoplasmatic tail. This protein is structurally similar to EBA-175 (25.3% aa identity) and EBA-140 (24.6%), especially in the F1-F2 domain (Gilberger et al., 2003).

The corresponding RBC receptor is the putative **“**W**”** receptor, which is susceptible to NM and chymotrypsin treatment (**Figure 2D** and **Table 1**) (Lanzillotti and Coetzer, 2006). It resembles a 110 kDa fragment from band 4.1 which is an important RBC membrane component providing mechanical resistance through vertical interaction with GPC and glycophorin D (GPD) and horizontal interaction with spectrin and actin (actin can destabilise the RBC**’**s cytoskeleton structure and inhibit membrane repair routes) (**Figure 3A**) (Lanzillotti and Coetzer, 2006).

Four HABPs have been identified when mapping EBA-181 binding to RBC: 30030 (^81^K-S^100^), 30045 (^381^D-K^400^), 30051 (^501^L-N^520^) and 30060 (^681^K-E^700^). Pre-treating RBCs with different enzymes (i.e. trypsin, chymotrypsin, NM, etc.) before carrying out the binding experiments has been used for characterising the chemical nature of the receptor. HABPs 30030, 30045 and 30051 fulfilled the protein**’**s enzymatic treatment profile described for the putative **“**W**”** receptor, being susceptible to NM treatment (reducing binding to RBC by 75%) whilst HABPs 30030 and 30051 were susceptible to chymotrypsin treatment of RBC (reducing binding by 92%). It was seen that HABP 30060 had the Plasmodium export element (PEXEL) motif (^683^RXK^685^LXF^687^S) contributing to protein transport through the membrane (Rodriguez et al., 2008; Patarroyo et al., 2016).

The *P. falciparum* Mrz invasion efficiency was not altered when glycophorin B-deficient erythrocytes were used (W2mefΔ181 transgenic lines), thus suggesting that EBA-181 is a redundant ligand and there are other parasite proteins with similar functions; this further confirms the complexity of the RBC invasion process and the ability of the parasite to use different invasion pathways (Gilberger et al., 2003).

#### 3.2.4 EBL-1 - Glycophorin B

EBL-1 was the second EBL family member to be identified, based on consensus sequence homology (Peterson et al., 1995). It is expressed during the schizont late stage as a truncated protein due to missense mutations; it has two Cys-rich domains in the N-terminal region DBL domain (D2), including subdomains F1 and F2, and a Cys domain adjacent to the C-terminal transmembrane region (Peterson and Wellems, 2000). The EBL-1 D2 domain or F2 region binds to RBC *via* the GPB receptor (**Figure 2D** and **Table 1**) which is resistant to trypsin and sensitive to chymotrypsin and NM treatment, indicating that it is a sialic-acid glycan epitope (Mayer et al., 2009; Li et al., 2012).

GPB is structurally similar to GPA, with the exception of the cytoplasmic tail which is much longer in GPA, and both are the main erythrocyte membrane sialo-glycoproteins. GPB is encoded by a separate gene that probably emerged by genetic duplication of the original GPA gene and is expressed as a 91 aa-long, ~10 kDa protein (having few copies per RBC: 20 to 100 x10^3^) (Li et al., 2012).

HABPs 29903 (^201^L-F^220^), 29923 (^601^C-V^620^), 29924 (^621^W-Y^640^) located in the DBL/F2 region and 30018 (^2481^L-Y^2500^) in the C-terminal region have been identified in a study of EBL-1 binding to RBC. HABP 30018 was extremely sensitive to RBC treatment with trypsin (reducing RBC binding by 65%) and chymotrypsin (by 95%). It has been reported that the EBL-1/GPB binding site is located in the F2i region (residues ^601^C-V^669^) where HABPs 29923 and 29924 have been located, suggesting that one or both could be involved in binding, making them promising antimalarial vaccine candidates (**Table 1**) (Curtidor, 2005; Li et al., 2012).

A level of redundancy has been proposed for EBA-175 and EBL-1 proteins that could bind to these similar glycophorins; GPB-negative RBCs have 40%-79% resistance to *P. falciparum* invasion and almost complete resistance after trypsin-treating RBC. It has been hypothesised that GPB may have arisen as a result of selective pressure to hamper *P. falciparum* **’**s main invasion route (Mayer et al., 2009, 1; Salinas et al., 2014).

#### 3.2.5 MAEBL-?

MAEBL is a 243 kDa type I membrane protein expressed in Mrz rhoptries and micronemes. It is considered an anomalous protein in the EBL family due to its Cys-rich domain resembling residues having apical membrane antigen-1 (AMA-1) instead of a DBL domain (i.e. being similar to AMA-1 I and II domains duplicated in tandem in M1 and M2 domains). MAEBL has a signal peptide (characterised by having a hydrophobic residue sequence) followed by a Cys-rich domain (M1/M2), a repeat in the central region, a transmembrane domain and a cytoplasmatic region (Ghai et al., 2002).

The MAEBL receptor on the RBC membrane has not yet been identified; however, it has been described that similar AMA-1 domains (M1 and M2) have high RBC binding ability. MAEBL HABPs identified to date have been 30195 (^401^T-H^420^) and 30198 (^461^Q-T^500^), located in M1 and susceptible to chymotrypsin treatment of RBC, and 30212 (^741^K-L^760^) and 30220 (^901^K-D^920^) located in the M2 domain (Rodriguez et al., 2008).

#### 3.2.6 PfRH1- Y

*Pf*RH1 has a high molecular weight (360 kDa), is 2,971 aa-long, and is expressed in the rhoptries. It undergoes proteolytic processing during invasion, becoming cleaved into 120 kDa and 240 kDa fragments; both are located in the TJ where the 120 kDa fragment is involved in parasite ring formation (Triglia et al., 2009).

Before EBA-175**’**s interaction with the GPA receptor, *Pf*RH1 binds to RBC *via* a 333 aa-long fragment (500-833) having a putative receptor called **“**Y**”** which is resistant to trypsin/chymotrypsin treatment and sensitive to NM (**Figures 2C, D** and **Table 1**) (Triglia et al., 2009). Abs targeting the RBC binding region could inhibit Mrz invasion (Gao et al., 2008).

HABPs 36381 (^441^D-H^460^), 36384 (^501^I-Y^520^), 36385 (^521^V-N^540^), 36389 (^601^I-K^620^), 36390 (^621^I-S^640^), have been identified for this protein (Arévalo-Pinzón et al., 2013). HABPs 36384, 36385, 36389 and 36390 lie within the RBC binding site described by (Triglia et al., 2009).

*Pf*RH1 plays a role immediately upstream of alternative route ligands by inducing Ca^2+^-dependent EBA-175 release followed by that of other rhoptry and microneme ligands, thereby inducing strong actin-dependent RBC membrane deformation. This has been suggested as mAb C41 has blocked Mrz invasion by ~55% by inhibiting the parasite’s Ca^2+^ signalling (Gao et al., 2013). This leads to Mrz reorientation, placing their apical pole perpendicular to RBC by means of EBL and *Pf*RH action during RBC invasion. As Mrz can use alternative RBC invasion pathways, *Pf*RH1 probably forms part of a more complex signalling network that translates Mrz binding to RBC surface into an intracellular Ca^2+^ signal.

#### 3.2.7 PfRH2a-? and PfRH2b-Z

*Pf*RH2a (370 kDa molecular weight) and *Pf*RH2**b** (383 kDa) are located in the rhoptry neck and are encoded by two contiguous genes in chromosome 13, sharing strong sequence similarity in the first 2,776 residues but then being divergent in the last C-terminal ~500 aa, which determine their different binding functions during the Mrz invasion (Dvorin et al., 2010).

*Pf*RH2a**’**s N-terminal region is cleaved into 90 kDa and 270 kDa fragments; 270 kDa is then cleaved into 130 kDa and 140 kDa fragments; these are transported to the TJ helping Mrz entry to RBC (**Figure 2D**) (Gunalan et al., 2011). The RBC receptor for *Pf*RH2a has not yet been characterised; however, HABP 26835 (^2761^L-Q^2780^) has been identified as being important in interaction with RBC (Rodriguez et al., 2008). Its functional relevance has been further confirmed since Abs targeting this protein can inhibit 3D7 strain parasite invasion by 40%–55% (Triglia et al., 2001).

*Pf*RH2b is cleaved into ~300, 250, 130 and 85 kDa fragments in W2-mef and 3D7 strains and is associated with *Pf*RH1, *Pf*RH4 and *Pf*RH5 in TJ formation during invasion to erythrocytes. It binds to RBC via putative receptor **“**Z**”** which is resistant to trypsin treatment and NM and sensitive to chymotrypsin (**Figures 2D** and **Table 1**) (Duraisingh et al., 2003b). HABPs 26529 (^3021^S-A^3040^) and 26534 (^3121^I-M^3140^) (having the ^3134^RT^3136^LD^3138^ PEXEL motif) have been described for this protein, being located in the C-terminal protein extreme (Rodriguez et al., 2008).

Alterations to the *Pf*RH2a and *Pf*RH2b gene do not produce differences regarding invasion phenotypes in knock-out or wild type parasites, suggesting that they are not essential for invasion, or that their function is compensated for by other parasite ligands (Duraisingh et al., 2003b). Interestingly, it has been found that Abs targeting *Pf*Rh2a/b (anti-Rh2a9) inhibited *P. falciparum* 3D7 growth by 35.6%. When EBA-175 and EBA-140 were absent (3D7Δ175/140) in presence of anti-Rh2a9, growth was inhibited by 60.5% (*p*=0.004), suggesting that *Pf*Rh2a and 2b compensate for the loss of EBA-175 and EBA-140 function in 3D7, this is consistent with overlapping roles that these protein families play during Mrz invasion, despite the exact mechanism remaining unknown (Lopaticki et al., 2011).

#### 3.2.8 PfRH4 – CR1

*Pf*RH4 is 1,716 aa-long and has a 206 kDa molecular mass. It interacts with complement receptor 1 (CR1) during invasion (**Figure 2D** and **Table 1**); CR1 is an immunoregulatory protein on RBC membrane and leukocytes (CR1 levels are genetically determined and associated with at least three single nucleotide polymorphisms in the CR1 gene). It is involved in complement system activation and the elimination of immune complexes (Tham et al., 2010).

The *Pf*RH4-CR1 interaction is resistant to NM treatment; this ligand is essential in sialic acid-independent invasion as disrupting the gene in the W2mef strain eliminates the parasite**’**s ability to change invasion routes and enables successful invasion of NM-treated RBC (Stubbs et al., 2005). Cell proliferation assays involving anti-*Pf*RH4 Abs have shown that it is a main ligand in the sialic acid-independent invasion route, depending on the strain (50-80%) (Tham et al., 2009).

Fine mapping of *Pf*RH4 interaction with RBC led to identifying HABP 34195 (^461^C-L^480^), 34203 (^621^Q-L^640^), 34215 (^861^L-I^880^), 34224 (^1041^Y-L^1060^), 34242 (^1410^L-Q^1420^) and 34243 (^1421^L-M^1440^) binding to RBC (García et al., 2010). The proteins**’** binding site was found between CR1 homologous repeat-A (LHR-A) (^18^D and F^20^) (Tham et al., 2010; Park et al., 2014) and a *Pf*RH4 30 kDa fragment (^328^N–D^588^) (Gaur et al., 2007) where HABP 34195 has been located (**Table 1**).

Alternative invasion route studies have shown that *Pf*RH4 resembles the main invasion ligand for NM-treated RBCs in the presence of soluble CR1 when using Mrz ΔEBA-175 (Δ means genetically deleted) (Tham et al., 2011). The invasion route becomes reduced nine fold when there is no alternative route. Weak RBC membrane deformation with ΔEBA-175 Mrz and NM-treated W2mef strain has been found, suggesting that *Pf*RH4 and EBA-175 have similar functions. EBLs/*Pf*RH interactions would thus seem to be responsible for strong RBC deformation during the pre-invasion stage (Lopaticki et al., 2011; Weiss et al., 2015). However, EBA-175 is the dominant ligand, its GPA receptor having around one million copies in RBC compared to 50-1,200 CR1 molecules per RBC.

#### 3.2.9 PfRH5 – Basigin

*Pf*RH5 is a 526 aa-long, rhoptry-located, 63kDa protein containing a signal peptide and an RH family homologous region (Baum et al., 2009). X-ray crystallography studies have shown that *Pf*RH5 adopts a flat, rigid structure formed by two domains, each mainly formed by three helices. The N-terminal domain begins with a short β-sheet followed by a short α-helix and two long α-helices connected by a truncated loop. The C-terminal domain is formed by three long helices covering the whole length of a domain terminating in a flexible C-terminus. The loop binding the structure is stabilised by a disulphide bond (^345^C-C^351^), whilst another disulphide bond (^224^C-C^317^) binds the second and third helices, leaving an antiparallel Cys (^329^C) (Wright et al., 2014).

*Pf*RH5’s crystal structure determined that it binds to BSG receptor type 2 (CD147) by the C-terminal extreme acting downstream of RBC membrane deformation. This interaction was found to be essential for RBC invasion by multiple *P. falciparum* strains (**Figure 2E** and **Table 1**) (Crosnier et al., 2011; Chen et al., 2014; Volz et al., 2016). BSG is a immunoglobulin superfamily transmembrane glycoprotein, is 385 aa-long and has a molecular weight of 42-66 kDa (depending on the amount of N-glycosylations) (Crosnier et al., 2011).

*Pf*Rh5 lacks a transmembrane domain and a GPI anchor signal, therefore, the mechanism by which it mediated RBC invasion without being anchored to the surface of the invading Mrz was not understood, until the complex it forms with *Pf*RH5-interacting protein (Ripr), cysteine-rich protector antigen (CyRPA) and P113 was described (detailed in the following section) (Chen et al., 2011; Reddy et al., 2015).

*Pf*RH5-BSG interaction triggers the release of rhoptry proteins facilitated by the flow of Ca^2+^ within the RBC and produces the phosphorylation of membrane skeleton proteins such as spectrin or band 3. Once phosphorylated, band 3 dissociates from ankyrin and spectrin, resulting in cytoskeleton weakening and its separation from the membrane at the entry site (Ferru et al., 2011, 3). The Ca^2+^ flux is inhibited during the parasite invasion in the presence of soluble BSG and anti-*Pf*Rh5, anti-basigin and anti-*Pf*Ripr Abs (Volz et al., 2016).

*Pf*RH5 HABPs 36727 (^201^G-V^220^), 36735 (^361^D-L^380^) and 36739 (^441^K-L^460^) have been found to be involved in binding to RBC. HABP 36727 had 61%-80% ability to inhibit parasite invasion, suggesting that it is a key HABP (Arévalo-Pinzón et al., 2012, 5). Contact residues between *Pf*RH5 and BSG C-terminal domain are located between aa 197-448 where HABPs 36727 and 36735 have been located (**Table 1**) (Arévalo-Pinzón et al., 2012; Wright et al., 2014).

One way in which this protein**’**s importance has been explored has been by using mAbs. Inhibiting *Pf*RH5 with an anti-*Pf*RH5 polyclonal IgG Ab resulted in a ~90% reduction of 3D7 strain invasion (Bustamante et al., 2013). No RBC re-invasion was observed when schizont rupture in the presence of this polyclonal Ab was monitored (Weiss et al., 2015). R5.016 has been reported as a potent anti-*Pf*RH5 mAb against *P. falciparum* strains FVO, Dd2, GB4, M-Camp, Cp845 and Cp806 (Alanine et al., 2019) from a panel of anti-*Pf*Rh5 mAbs isolated from a phase I clinical trial of a *Pf*Rh5-based vaccine (Payne et al., 2017). Its EC_50_ occurred at 9.6 μg/mL, ​​comparable with that for potent anti-Mrz mAbs 9AD4 (neutralising parasite RBC invasion by 70%), QA1 (38%), QA5 (63%) and 6BF10 (30%) (Douglas et al., 2014).

It has been shown that *Pf*RH5 has limited polymorphisms and provokes potent neutralising Abs inhibiting Mrz invasion of RBCs, making it an attractive blood stage vaccine candidate (Reddy et al., 2015; Payne et al., 2017; Alanine et al., 2019).

#### 3.2.10 PfRipr – Semaphorin-7A

*Pf*Ripr is a 123 kDa protein, containing 87 cysteines and 10 epidermal growth factor-like domains that are processed into 2 polypeptides that associate and form a complex with *Pf*Rh5 (Chen et al., 2011).

*Pf*Ripr is located in the Mrz micronemes and is released during RBC invasion and forms a ternary complex with *Pf*RH5 and CyRPA (Chen et al., 2011; Reddy et al., 2015). CyRPA is a 43 kDa protein located in Mrz micronemes (Ragotte et al., 2020). Transmission electron cryomicroscopy (CryoTEM/cryo-EM) has suggested that CyRPA is the core stabilising *Pf*Rh5 and Ripr on the opposite sides of the ternary complex, following *Pf*Rh5-BSG binding. The complex thus becomes disassembled and CyRPA becomes excluded from the membrane whilst *Pf*RH5 and Ripr are inserted into it, thereby enabling the formation of pores in RBC membrane through which Ca^2+^ enters cells (Wong et al., 2019). However, a recent report has described a direct *Pf*Ripr-*Pf*Rh5 K_D_ 4.8 x 10^-10^ M and K_a_ 6.1 x 10^4^ (1/Ms) interaction; anti-*Pf*Ripr_5 (^720^C-D^934^) Ab have inhibited such an interaction by 25 ± 5% (*p*<0.05) (Nagaoka et al., 2020).

Like *Pf*Rh5, *Pf*Ripr and CyRPA lack a GPI anchor which, again, makes it difficult to explain their anchor mechanism during RBC invasion. It has been described that P113 (a GPI-anchored protein) binds to *Pf*RH5 N-terminal domain *via* a 19 aa (^9^K-K^27^) stretch. P113 also interacts with CyRPA but is not associated with *Pf*Ripr or BSG (**Figure 2E**) (Galaway et al., 2017, 5).

*Pf*Ripr interacts with RBC receptor semaphorin-7A (Sema-7a) (**Table 1**) (Nagaoka et al., 2020). Sema-7a is a 666 aa-long glycosylated membrane protein and is associated with cell surfaces through the glycosylphosphatidyl-inositol (GPI) domain. This protein is expressed in RBCs, pre- and post-natal neurons, lymphocytes and mitogen-activated thymocytes and is involved in axon guidance during neurogenesis and molecular and physiological processes, such as vascular growth, organogenesis, regulation of immune cells by associating with integrins and inflammatory cytokine production (Xie and Wang, 2017). This interaction was inhibited by anti-*Pf*Ripr_5 mAb (^720^C-D^934^) by 58% ± 3% (*p*<0.05) (Nagaoka et al., 2020). It has also been observed *in vitro*, in invasion inhibition assays, that recombinant *Pf*Ripr inhibited parasite growth by 21% ± 4% (Nagaoka et al., 2020), indicating this protein’s significant role during parasite invasion of RBC.

Anti-*Pf*Rh5, -CyRPA and/or -Ripr Ab mixtures have acted additively and synergistically during *in vivo* and *in vitro* assays; a vaccine candidate including a mixture of these antigens would thus be expected to be highly protective (Favuzza et al., 2017; Healer et al., 2019; Illingworth et al., 2019). It has been described that specific anti-*Pf*Rh5, non-inhibitory mAbs can synergise with inhibitory anti-*Pf*Rh5, CyRPA and *Pf*Ripr mAbs by slowing down parasite RBC invasion 3-fold (Alanine et al., 2019).

Developing a vaccine against the *Pf*Rh5-CyRPA-Ripr-P113 protein complex (binding RBC through BSG and Sema-7a) would be promising due to the high degree of aa sequence conservation and the lack of redundant pathways that can substitute its function (Ragotte et al., 2020).

### 3.3 Tight Junction Formation

#### 3.3.1 AMA-1 – RON

Mrz’s irreversible binding to RBC is produced by TJ formation, this being shaped from the Mrz´s apical to posterior end in a complex series of events supported by high affinity interactions involving rhoptry neck proteins (RON-2, -4 and -5). These are released from the rhoptries and translocated to the RBC membrane to interact with AMA-1 (**Figure 2F**) (Alexander et al., 2006).

AMA-1 is a low-abundance type I integral membrane protein, synthesised as an 83 kDa polypeptide located in the micronemes. It is formed by a large ectodomain, divided into a pro domain and three domains (DI, DII, and DIII), followed by a transmembrane helix and a cytoplasmic C-terminal tail. The crystal structure of AMA-1 has shown that the receptor-binding site includes a hydrophobic pocket and a region that becomes exposed by displacement of the flexible DII loop (Vulliez-Le Normand et al., 2012).

RON-2, RON-4 and RON-5 form a complex released from Mrz rhoptry organelles prior to penetration before becoming embedded on RBC surface. RON-4 and RON-5 are located on RBC membrane cytoplasmic face whilst RON-2 contains several internal transmembrane domains and extends on both sides of the membrane, presenting a surface-exposed, long β-hairpin loop on the C-terminal domain which interacts with AMA-1’s hydrophobic groove. The β-hairpin loop binds to this groove and displaces the DII loop, exposing a new surface to the surrounding environment (Wang et al., 2016, 0); mAb 4G2, binding near the hydrophobic pocket, and/or mAb 1F9, binding directly to it, interfere with *P. falciparum* Mrz invasion (Collins et al., 2007; Collins et al., 2009).

*P. falciparum* RON2 conserved region, called RON2L (DITQQAKDIGAGPVASCFTTRMSPPQQICLNSVVNTALSTSTQSAMK), has two completely conserved cysteines in its sequence which have a structural function providing proper binding region orientation (Srinivasan et al., 2011). AMA-1-RON2sp1-derived peptide (RON-2 aa 2,021-2,059) crystal structure has shown that the RON-2 C-terminal domain β-hairpin loop has a highly conserved disulphide bridge between C^2037^ and C^2049^ which is important for stabilising β-hairpin conformation. A *cis*-Ser-Pro segment located at the tip of the loop produces a reversal of the β-hairpin to maintain a unique loop conformation for binding to AMA-1. It has been revealed that AMA-1 F^2038^ and R^2041^ are important in AMA-1-RON-2 interaction because its side chains interact with a shallow pocket in the centre of the hydrophobic groove (Wang et al., 2016). RON2L and AMA-1 interaction has been inhibited by adding mAbs 1F9 (~90%) and 4G2 (~60%); 0.5-2 mg/mL of anti-RON2L Abs has blocked RBC invasion (~10%-90%) by 3D7 and FVO strains (Srinivasan et al., 2011).

Several studies have focused on inhibiting *P. falciparum* invasion using Abs directed against AMA-1, preventing Mrz reorientation or TJ formation, suggesting that AMA-1 and RON-2 interactions play an important role in invasion (Hodder et al., 2001; Kennedy et al., 2002; Healer et al., 2004). However, it has been described that Mrz protein release occurs even in the absence of the AMA1-RON-2 complex; it is therefore considered that the *Pf*RH5-BSG interaction could be responsible for triggering the release of Mrz rhoptry proteins which are apically orientated to the RBC (Weiss et al., 2015). Supporting the above, FMP2.1/AS02a (an AMA-1-based recombinant protein malaria vaccine candidate) has conferred partial strain specific efficacy during field trials due its polymorphic nature (Thera et al., 2011), suggesting that it might be useful in a multicomponent vaccine.

AMA-1 domain III interaction with RBC membrane protein Kx has been described (resistant to trypsin and neuraminidase treatment) (**Table 1**). The host protein Kx is 440 aa-long, traverses the RBC membrane ten times and carries the Kx blood group antigen. Its covalent binding to the Kell protein constitutes the Kell blood group system; these are linked by a single disulphide bond between Kell Cys^72^ and Kx Cys^347^ and are mainly expressed in erythroid tissues (Lee et al., 2000). RBC Δ Kx invasion rate becomes reduced, indicating this protein’s significant role in parasite invasion (Kato et al., 2005).

#### 3.3.2 MTRAP – Semaphorin-7A

Successful invasion requires a single-headed myosin adhered to the RBC membrane for uni-directionally driving short actin filaments to impart motive force. The actin filaments are coupled to invasins which belong to the thrombospondin-related anonymous protein family (TRAP). TRAP-family proteins are believed to interact with host receptors through the extracellular regions, thus activating the actin-myosin motor during the parasite’s forward movement (Bartholdson et al., 2012).

*P. falciparum* merozoite-specific TRAP homologue (MTRAP) is a 45 kDa protein which acts as a bridge between the motor and the inner RBC membrane. Its ectodomain interacts with Sema-7a (**Figure 2G** and **Table 1**) (Bartholdson et al., 2012). It has been observed in *in vitro* invasion inhibition assays that Abs against Sema-7a have not inhibited Mrz invasion of RBCs (Bartholdson et al., 2012), this indicates that MTRAP is not an essential *P. falciparum* ligand and that the parasite can use several other RBC invasion routes.

Cross-linking assays have identified HABP 33405 (^21^Y-H^40^) in the N-terminal region containing a PEXEL-like sequence and 33413 (^180^L-Y^199^) in the protein’s central region. HABP 33405 bound to a ~72 kDa RBC protein (Sema-7a ~74 kDa). Trypsin, chymotrypsin and NM treatment did not have any effect on HABP 33405 binding to RBCs, whilst they produced marked sensitivity for HABP 33413 (Calderón et al., 2008).

The complex interactions between MTRAP – Sema-7A and AMA-1 – RONs form a stable, high avidity ring-like structure that serves as an anchor providing the necessary pulling machinery for Mrz to enter the PVM within RBC, together with the parasite’s submembrane actin-myosin motor (**Figures 2G, H**) (Yap et al., 2014).

Furthermore, MTRAP is essential for the rupture of the PVM that contains male and female gametes, thereby enabling them to exit the iRBC and promote parasite transmission to female *Anopheles* mosquitos (Bargieri et al., 2016).

All the above multi-step processes occur within about 60 seconds, an efficient but critical timeframe because it represents a short period in the parasite’s lifecycle when directly exposed to a host’s immune system. The immunogens used to inhibit such complex parasite invasion must thus induce the production of abundant, highly specific Abs.

It is worth highlighting that, despite great efforts made to elucidate the protein-protein interactions between *P. falciparum* and RBC, just 10 receptors have been characterised for the more than ~50 described parasite proteins. Interestingly, nine of the ten host cell receptors described so far express blood group antigens as a common feature (**Table 1**). Some plasma membrane proteins carrying blood group antigens also play an important role during Mrz invasion, such as complement decay-accelerating factor (CD55 antigen), extracellular matrix receptor III (CD44 antigen), the Kell blood group glycoprotein, intercellular adhesion molecule-4 (ICAM-4), membrane glycoprotein SFA-1 (CD151) and small integral membrane protein 1. However, their specific parasite binding ligands are presently unknown (**Table 2**) (The UniProt Consortium, 2021).

**Table 2:** Potential *Plasmodium falciparum* RBC receptors.

RBC membrane protein	Blood group system expressed	Molecular mass (kDa)	Length(aa)	Molecular functions and biological process	Relationship with *P. falciparum* entry to RBCs	Ref
Complement decay-accelerating factor (CD55)	Cromer	41	381	- Lipid binding. - Complement activation	Using gene knockdown, RBCs deficient in either CD55 or CD44 were produced; ~30% reduction in the parasitaemia were observed with either knockdown.	(Egan et al., 2015)
Extracellular matrix receptor III antigen (CD44)	Indian	81	742	- Collagen and hyaluronic acid binding. - Transmembrane signaling receptor activity. - Cell adhesion. - Extracellular matrix organisation	CD44 was deleted using CRISPR/Cas9, producing a strain-transcending reduction in parasite invasion (~40%).	(Kanjee et al., 2017)
Kell blood group glycoprotein	Kell	82	732	- Metal ion binding. - Cellular calcium and magnesium homeostasis. - Regulation of cell size	Kell is a type II glycoprotein co-expressed with Kx (*Pf*AMA-1 receptor). By blocking Kell with a specific Abs, *P. falciparum* invasion of RBCs was reduced by ~50%.	(Pati et al., 2020)
ICAM-4	Landsteiner-Wiener	29,2	271	- Integrin binding. - Cell-cell adhesion. - Extracellular matrix organization - Regulation of immune response	The M-5 protein was identified using ‘codon-shuffled’ synthetic libraries, and it bound to ICAM-4 (KD 10.07). Purified schizont-stage parasites (3D7 strain), were incubated with different concentrations of M-5, decreasing the parasitaemia with a maximum of ~80%. A similar behaviour was observed when RBCs infected with either Dd2 or MCamp (sialic acid-dependent strains) or HB3 (sialic acid-independent strain) were pre-incubated with M-5.	(Bhalla et al., 2015)
Membrane glycoprotein SFA-1(CD151)	RAPH	28,2	253	- Integrin binding. - Cell adhesion. - Positive regulation of cell migration.	Inhibition assays using purified anti-CD151 immunoglobulin G from rat or rabbit in RBCs infected with *P. falciparum* trophozoite stages (FCR3 strain) or isolates from patients with severe malaria, showed statistically significant parasite inhibition in a dose-dependent manner (~30%).	(Esser et al., 2014)
Small integral membrane protein 1	Vel	8,7	78	- Signaling receptor binding. - Regulation of cytokine production. - T cell receptor signaling pathway.	The small integral membrane protein 1 is a non-glycosylated molecule. After *P. falciparum* invasion of RBCs, the protein is phosphorylated during the schizont stage, however, it has not been associated yet with any function during parasite invasion. Being a small protein, it would not be unusual for it to be part of a functional protein complex.	(Solyakov et al., 2011)

### 3.4 Active Invasion

The parasite enters the RBC by a moving junction and is located in the PVM; this vacuole is useful for isolating itself from a host cell’s cytoplasm and forming an environment conducive to its development (**Figure 2H**) (Weiss et al., 2016). There is controversy regarding whether the PVM is derived from the parasite, from host material or both, and its small size represents technical difficulties for specific isolation of PV content for proteomic analysis.

Phosphatase *Pf*shelph2 is released as soon as Mrz invade the RBC; this dephosphorylates band 3 using a divalent metal cation-dependent catalytic mechanism causing erythrocyte cytoskeleton re-sealing and restoration, thereby helping to initiate parasite maturation in host cells (Fernandez-Pol et al., 2013).

The parasite remains in its ring form during the first 18 hours of its lifecycle, having low metabolic activity and changes little morphologically regarding host cells. The parasite then matures into its trophozoite form (feeding stage) in which it becomes more metabolically active and grows rapidly. DNA synthesis and nuclear division occurs during the schizont stage to form 16-32 Mrz **(** **Figures 2H–K**) (Cowman et al., 2017). This is followed by rapid cytokinesis enabling the budding of individual nuclei and the organelles required for invasion to form individual Mrz. These are released from infected iRBC (during a process known as egress) to invade new RBC and continue the blood cycle (**Figure 2L**) (Weiss et al., 2015).

## 4 Remodelling Red Blood Cells for Parasite Survival

*P. falciparum* exports a substantial number of proteins to acquire nutrients, modify iRBC membrane properties and avoid the immune system to promote its own survival within the PVM. Once the exported proteins cross the PVM they are transported from the cytoplasm to the iRBC membrane by novel parasite-derived membrane structures, such as the tubovesicular network (TVN), Maurer’s clefts, K-dots, J-dots and exosome-like vesicles that work as a sorting point from which parasite proteins are deposited underneath or into the iRBC membrane (**Figures 2H–K**) (Jonsdottir et al., 2021).

The TVN forms an intricate network extending from the PVM to host cell periphery, penetrating deep into the iRBC but does not appear to fuse with the RBC membrane (**Figures 2I–K**). There is controversy as to whether the TVN is encoded by parasite proteins or results from RBC protein modifications (Alkhalil et al., 2004; Huber et al., 2005). The TVN provides efficient access for acquiring extracellular nutrients, including sugars, aa, purines, vitamins, choline and fatty acids. The TVN also facilitates the removal of parasite metabolic waste products (Martin and Kirk, 2007). Pharmacologically inhibiting the TVN could lead to nutrient deprivation and toxic metabolite accumulation, leading to parasite death.

Maurer’s clefts are membrane vesicles in iRBC cytoplasm or attached to the membrane skeleton; they are important in the iRBC remodelling due to protein sorting and repackaging in vesicles for traffic (**Figures 2H–L**). These membrane structures are important regarding immune evasion; gene-knockout studies eliminating Maurer’s cleft proteins, such as MAHRP1 and SBP1, have resulted in *Pf*EMP1 not being exported to iRBC surface, reduced cytoadherence levels and host cell rigidity (Cooke et al., 2006; Maier et al., 2007).

In a recent study, the ΔGEXP07 parasite line was generated by CRISPR-Cas9 and homology-directed repair, the absence of this protein produced Maurer’s cleft fragmentation, aberrant knobs, ablation of *Pf*EMP1 surface expression, and loss of the *Pf*EMP1-mediated adhesion (McHugh et al., 2020). This suggests that the membrane structures formed by parasite invasion of RBC thus represent an attractive target for developing antimalarial drugs.

## 5 Nutrient Acquisition and Induction of New Permeability Pathways

*P. falciparum* matures rapidly in the RBC, requiring a vast range of essential nutrients; however, host cells cannot provide them because they are devoid of intracellular organelles, lacking *de novo* protein synthesis and endogenous protein trafficking machinery. Hence, many of the critical nutrients such as sugars, anions, cations, pantothenate, choline, isoleucine, glutamine, glutamate, methionine, proline, tyrosine, cysteine, hypoxanthine, purine precursors and organic solutes required by the parasite can be transported *via* inherent RBC membrane transporter activity and be subsequently used by the Mrz. However, others are not transported or transported in such low amounts that are not enough to sustain rapid parasite maturation; the parasite therefore increases iRBC permeability and induces new, parasite-derived permeability pathways (NPP) to transport them from iRBC cytosol and the extracellular milieu (**Figure 3B**) (Martin and Kirk, 2007; Gilson et al., 2017).

However, if the iRBC membrane becomes highly permeable, high haemoglobin concentrations within the cells will cause osmotic entry of fluids and cell swelling and bursting. The parasite thus degrades haemoglobin to avoid early bursting of the iRBC and uses it as its main source of aa. Different proteases have been described regarding haemoglobin digestion, including the aspartic proteases (plasmepsin I, II, III, IV), cysteine proteases (falcipain-1, falcipain-2, falcipain-3) and aminopeptidases (Belete, 2020). A study has shown that different cysteine protease inhibitors could inhibit *P. falciparum* growth and haemoglobin degradation, thus being promising anti-malarial targets (Glushakova et al., 2009).

Different anion channels appear on iRBC some hours after parasite invasion; one such parasite-encoded ion channel is the plasmodial surface anion channel (PSAC) formed by *P. falciparum* CLAG3, RhopH2 and RhopH3 (**Figure 3**) (Jonsdottir et al., 2021). According to recent studies based on Förster resonant energy transfer (FRET) and cryo-electron microscopy, the probable function of RhopH2 and RhopH3 is to escort CLAG3’s soluble form to the iRBC surface where CLAG3 can then insert itself into the host cell membrane (Ahmad et al., 2020; Schureck et al., 2021). PSAC represents one of the most promising anti-malarial targets due to its critical function concerning nutrient acquisition (Belete, 2020).

## 6 Loss of Deformability and Adhesion

The RBC membrane adopts an echinocyte shape seconds after the Mrz have completely entered the host cell. This morphological change is characterised by the curling of the edges of the biconcave discoid cells, which advances to the formation of spicules, and then a return to their normal discocyte shape after a few minutes. The consequences of echinocytosis are not known; they may be related to plasma membrane resealing following Mrz entry, PVM maturation and/or the creating of Maurer’s clefts (Mbengue et al., 2012).

The iRBC begin to lose their biconcave shape after Mrz invasion and become progressively spherical, rigid, and their surface-volume ratio decreases. In addition to the loss of deformability, iRBC adhesive properties promote cytoadherence to the microvasculature endothelium, sequestration and vascular occlusion; this leads to accumulations of iRBC in different organs, avoiding surveillance by and clearance in the spleen (**Figure 1G**) (Li et al., 2018).

Different *P. falciparum*-derived variant surface antigen (VSA) groups have been described as being involved in iRBC adhesion, including *P. falciparum* erythrocyte membrane protein 1 (*Pf*EMP1) (Chen et al., 1998), subtelomeric variable open reading frame (STEVOR), (Niang et al., 2014) and repetitive interspersed family (RIFIN) (Goel et al., 2015) (**Figure 2J**, **3B**). In order of parasite maturation, *Pf*EMP1 and RIFIN are expressed on iRBC surface during the early trophozoite stage, followed by STEVOR which appears during the late trophozoite stage (Yam et al., 2017).

*Pf*EMP1 is a key protein in the pathophysiology of *P. falciparum* due to antigenic variation, adherence functions, and immune evasion properties. The protein is encoded by the *var* multigene family which provides the parasite with 60 serologically diverse adhesins having different binding characteristics (Smith et al., 2013). The protein is exposed to the host’s immune system due to its surface location; however, the parasite can switch the *var* gene being expressed, thereby enabling clonal antigenic variation to evade an immune response (Hviid and Jensen, 2015; Moxon et al., 2020).

*Pf*EMP1 contains a polymorphic extracellular domain, linked through a single transmembrane helix to a relatively conserved cytoplasmic domain. The ectodomain comprises the N-terminal segment, the C2 region, the Cysteine-rich inter-domain (CIDR) characterised by conserved cysteine-rich motifs and the DBL region, a domain which belongs to the parasite’s adhesion-domain superfamily EBLs (Hviid and Jensen, 2015).

*Pf*EMP1 is exported to the iRBC membrane and is directly linked to the knob-associated His-rich protein (KAHRP). *Pf*EMP1’s cytosolic domain largely interacts with spectrin and KAHRP has been associated with β-spectrin domains 10–14. The *Pf*EMP1- KAHRP interaction assists in the formation of knobs (electron-dense protrusions) covering the surface of late parasite stage iRBC, representing adherent contact points with host receptors (**Figures 2I–L**, **3B**) (Cutts et al., 2017). *Pf*EMP1 trafficking to the knobs is associated with severe adhesion-related pathologies such as placental and cerebral malaria (Miller et al., 2002).

The *Pf*EMP1 extracellular domain interacts with varied host receptors and mediates iRBC cytoadherence to endothelial cells (**Figures 2J**, **3B**). The iRBC have different binding properties varying between clones and each *Pf*EMP1 can bind to several receptors in different organs through the DBL and CIDR1 domains (**Table 3**) (Yipp et al., 2000; Vogt et al., 2003, 1; Vigan-Womas et al., 2012; Kessler et al., 2017). STEVOR interacts with GPC and RIFIN with blood group A antigen and GPA; these receptors are mostly located on RBC while *Pf*EMP1 receptors are also found on endothelial cells (**Table 3**) (Niang et al., 2014; Goel et al., 2015).

**Table 3:** *Pf*EMP1, STEVOR and RIFIN interactions with host cell receptors.

Ligand	Host cell receptor	Receptor expression site
*Pf*EMP1	Platelet glycoprotein 4 /CD36	Endothelial and epithelial cells, monocytes, platelets, macrophages, erythrocyte precursors, skeletal muscles and adipocytes
	PECAM-1	Endothelial cells, monocytes, platelets, neutrophils, T-cells and granulocytes
	E-selectin	Endothelial cells
	ICAM1	Endothelial cells and leukocytes
	VCAM	Endothelial cells
	P-selectin	Activated platelets and endothelial cells
	Complement receptor 1	Neutrophils, monocytes, B-lymphocytes, RBCs and splenic follicular dendritic cells
	NCAM	Endothelial cells
	Chondroitin sulphate A	Endothelial cells and are abundant within the placental intervillous space
	Hyaluronic acid	Placenta, epithelial and neural tissues
	Heparan sulphate	All tissues
	IgM	Circulation
	Endothelial protein C receptor	Endothelial cells and leukocytes
	Hyaluronan-binding protein 1	Extracellular matrix, endothelial cells and platelets
	A or B group antigens	RBCs
RIFIN	RBC group A antigen	RBCs, B cells and NK cells
	GPA	RBCs
STEVOR	GPC	RBCs

These proteins’ interaction can produce late-stages iRBC binding to uRBCs through interactions with different host receptors forming clusters of cells called rosettes (**Figure 1G**). Extensive parasite rosetting pathways suggest that there has been significant selection pressure and that rosetting formation could improve *P. falciparum* fitness (McQuaid and Rowe, 2020). However, rosetting’s biological role remains unknown; it has been attributed to different functions such as parasite survival providing close uRBC to ensure rapid Mrz re-invasion and masking strategy proposing that uRBC mask the VSAs expressed on iRBC surface and thus evade host immune system recognition (Wahlgren et al., 1989).

Increased membrane rigidity and adhesion properties lead to sequestration and vascular occlusion, mediating the obstruction of microvascular blood flow, one of the most important pathological events in severe malaria. This leads to localised endothelial dysfunction, ischemia, tissue damage and organ failure by damaging endothelial barrier integrity and inducing pro-inflammatory, pro-adhesive and coagulation pathways.

## 7 Protein Trafficking

*P. falciparum* exported proteins have to cross the parasite’s plasma membrane and the PVM to access iRBC cytosol; the exported proteins have a signal sequence in the N-terminal which gives them the trafficking direction (Mbengue et al., 2012). The proteins exported into the PVM have a hydrophobic signal peptide and proteins transported beyond the PVM have the PEXEL motif, also called vacuolar transport signal (VTS), which consists of a conserved sequence of five aa (RxLxE/Q/D) where x represents any charge-neutral aa (Schulze et al., 2015).

The PEXEL motif is located 25-30 aa downstream of the endoplasmic reticulum (ER) signal sequence (de Koning-Ward et al., 2009). Exported proteins are cleaved after the conserved leucine (L) residue by proteases in the ER and subsequently N-acetylated, enabling protein translocation by ATP-driven translocon (called the PTEX complex) which is associated with parasite-derived membrane structures to transport the proteins to the iRBC cytoplasm or membrane (Boddey et al., 2009). Characterising the conserved motif has enabled creation of the *in silico* PEXEL exportome databases; the last one published included 463 proteins. However, the function for most of them remains unknown (Boddey et al., 2013). Large-scale gene knockout screening showed that ~25% of the exported proteins are essential for parasite survival (Maier et al., 2008), ~13% are essential for Mrz replication *in vitro* (Jonsdottir et al., 2021) and some of them promote infected cell adhesion and rigidity (Dhangadamajhi et al., 2010).

Exported proteins differing from the PEXEL conserved pattern have been found, for instance, those having an additional charge-neutral aa in the fifth position (Boddey et al., 2009; Boddey et al., 2013). An increasing number of PEXEL-negative exported proteins (PNEP) are exported into the iRBC, such as skeleton binding protein 1 (*Pf*SBP1) related to Maurer’s clefts, skeleton binding protein 1 (SBP1), membrane-associated histidine-rich protein 1 (MAHRP1) and the ring-exported protein (REX) (Dhangadamajhi et al., 2010), indicating that the parasite has different PEXEL-independent pathways to export proteins.

## 8 Parasite Egress from Host Cells

The last stage of *P. falciparum* development in the RBC is the release of multiple Mrz from host cells, a rapid (400 milliseconds) and necessary process for inducing new cycles of intraerythrocytic development and maintaining Mrz proliferation. Once most of the haemoglobin has been consumed, mature schizont rupture and a new generation of 8-24 Mrz egress, passing through the disrupted PVM, the host cytoskeleton and iRBC membrane rupture and vesiculation (i.e. the ‘inside-out’ model) (Tan and Blackman, 2021). Every RBC lysis releasing mature Mrz results in exponential growth and rapid disease progression, producing fever and severe anaemia (**Figures 1D**, **2L**) (Singh and Chitnis, 2017; Moxon et al., 2020).

Mrz egress is a synchronic and multi-step event produced by osmotic stress and iRBC elastic instability (Abkarian et al., 2011). A few minutes before the *P. falciparum* Mrz release, the RBC swell and the use of amphiphiles, osmotic stress and protease inhibitors have strongly suggested that parasite release is pressure-driven (Blackman, 2008; Mbengue et al., 2012; Singh and Chitnis, 2017).

Guanylyl cyclases (GCs) synthesise cyclic GMP (cGMP) and (along with cyclic nucleotide phosphodiesterases) regulate intracellular messenger levels mediating many eukaryote functions (Nofal et al., 2021). *P. falciparum* protein kinase (PKG) is activated by the nucleotide cyclic guanosine monophosphate (cGMP); its regulation levels control parasite egress from iRBC. Exit time can only be mediated by cGMP accumulation in the parasite until it reaches the critical intracellular concentration required to activate PKG (Tan and Blackman, 2021).

Parasite protein phosphatase 1 (*Pf*PP1) plays an essential role in GCα activity (Paul et al., 2020). PKG activation results in inositol (1,4,5)-triphosphate (IP3) production and intracellular Ca^2+^ mobilisation (Brochet and Billker, 2016). This activates Ca^2+^-dependent protein kinases (CDPKs) and triggers specialised secretory organelle discharge; CDPK5 is required for efficient egress, cooperating with PKG in a poorly-understood manner for controlling parasite egress (Absalon et al., 2018). It has been reported that the blockade of cGMP synthesis by conditional inactivation of guanylyl cyclase GCα blocks parasite egress from iRBC (Nofal et al., 2021).

The parasite’s subtilisin-like serine protease SUB (*Pf*SUB1) is a critical secretory organelle for Mrz and gamete egress; this is activated by the aspartic protease plasmepsin X (PMX) (Nasamu et al., 2017; Pino et al., 2017). *Pf*SUB1 produces MSP1 proteolysis by altering its secondary structure; SUB1-processed MSP1 interacts with heparin and spectrin, located in iRBC cytoplasm, helping to regulate cell rupture and parasite release (Das et al., 2015). Likewise, *Pf*SUB1 interacts with soluble papain-like PV proteins from the Serine Repeat-Antigen (SERA) family, involved in the egress of the parasite from iRBCs, hepatocytes and mosquito stages. The events downstream of *Pf*SUB1 activation and MSP/SERA processing are less well understood (Hale et al., 2017). Aspartic proteases, plasmepsins IX (PMIX) and PMX, block parasite egress and prevent Mrz protein maturation required for RBC invasion (Nasamu et al., 2017; Pino et al., 2017), thereby constituting a promising malaria drug target (Favuzza et al., 2020).

Before Mrz egress from iRBCs, an intracellular Ca^2+^ increase has been detected in schizonts, probably regulated by the secretion of perforin-like protein 1 (*Pf*PLP1) from Mrz micronemes; intracellular Ca^2+^ chelation has been shown to inhibit *Pf*PLP1 secretion, thus hampering parasite egress (Garg et al., 2013).

There is growing interest in investigating mechanisms aimed at blocking parasite egress from the iRBC to stop mature Mrz release and to interrupt the parasite’s lifecycle. Cysteine and serine protease inhibitors, such as E-64, block PVM disruption and leupeptin and antipain inhibit iRBC membrane disruption. However, their targets are not yet known (Glushakova et al., 2009).

## 9 Concluding Remarks

*P. falciparum* entry to RBC is an essential component of its life-cycle. RBC invasion is an intricate multi-step process mediated by the specific interactions between parasite ligands and RBC receptors. The parasite is able to use multiple invasion routes to achieve entry, being able to express several, redundant ligands to invade the RBC despite host immune-mediated selection or the RBC receptors’ genetic polymorphisms in malaria-endemic areas. However, knowledge regarding the membrane receptors on RBCs and the various routes used by *P. falciparum* to invade host cells is very limited. About 50 parasite ligands have currently been characterised and just 10 membrane receptors, i.e. band 3, GPA, GPB, GPC, band 4.1, CR1, BSG, Sema-7A, Kx, heparin and putative receptors “W” “Z” and “Y”. Elucidating specific receptor-ligand interactions will contribute to knowledge regarding the parasite’s biology and developing prophylactic and/or therapeutic measures that could help mitigate this scourging disease.

As soon as the parasite succeeds in entering host cells it is faced by another obstacle, i.e. surviving in RBC devoid of intracellular organelles, lack of *de novo* synthesis and endogenous protein trafficking machinery. The parasite increases iRBC permeability to overcome such challenges and induces membrane structure formation to obtain enough nutrients from the iRBC cytosol and the extracellular milieu to grow and survive. The parasite also promotes cytoadherence to microvasculature endothelium, sequestration and vascular occlusion, thereby producing accumulations of iRBCs in vital organs, avoiding surveillance and clearance by the spleen. Once the parasite matures, it is released from host cells and the cycling and destruction of human erythrocytes is repeated, producing anaemia which is characteristic of the disease.

Surprisingly, little research has been devoted to elucidating the mechanisms by which the parasite creates membrane structures, modifies RBC membrane properties and creates NPP to acquire nutrients, remove waste products and promote cytoadherence to evade an immune response. Developing anti-malarial mechanisms aimed at interfering with them would prevent nutrient uptake by the parasite, producing stress during its maturation and obstructing recognition by the host’s immune system through cytoadherence.

All the above cellular and molecular mechanisms involved in parasite invasion, growth, multiplication and final release make *P. falciparum* the most lethal malarial species. There is great interest in elucidating and fully understanding such molecular mechanisms driven by the urgent need to develop prophylactic and/or therapeutic measures helping to mitigate malaria, a worldwide public health problem. Current anti-malarial vaccine candidates have had limited efficacy due to malaria’s inherently complex lifecycle (Salamanca et al., 2019; Molina-Franky et al., 2020). A truly effective anti-malarial vaccine has to include multiple conserved epitopes against the several parasite proteins used in the multiple invasion routes (multi-epitope) and cover the different parasite forms (multi-stage) to induce strain-transcending protection and overcome the parasite’s genetic variability.

## Author Contributions

JM-F conceived the work, drafted the manuscript and designed the figures. MEP, MK and MAP helped conceive the work and critically revised the manuscript. All authors have revised the manuscript and given their approval for this version to be submitted.

## Funding

This project was funded by the Department of Immunology and Theranostics of the City of Hope and the Fundación Instituto de Inmunología de Colombia (FIDIC).

## Conflict of Interest

The authors declare that the research was conducted in the absence of any commercial or financial relationships that could be construed as a potential conflict of interest.

## Publisher’s Note

All claims expressed in this article are solely those of the authors and do not necessarily represent those of their affiliated organizations, or those of the publisher, the editors and the reviewers. Any product that may be evaluated in this article, or claim that may be made by its manufacturer, is not guaranteed or endorsed by the publisher.
